# Attention-deficit/hyperactivity disorder and occupational outcomes: The role of educational attainment, comorbid developmental disorders, and intellectual disability

**DOI:** 10.1371/journal.pone.0247724

**Published:** 2021-03-17

**Authors:** Andreas Jangmo, Ralf Kuja-Halkola, Ana Pérez-Vigil, Catarina Almqvist, Cynthia M. Bulik, Brian D’Onofrio, Paul Lichtenstein, Ewa Ahnemark, Tamara Werner-Kiechle, Henrik Larsson

**Affiliations:** 1 Department of Medical Epidemiology and Biostatistics, Karolinska Institutet, Stockholm, Sweden; 2 Department of Child and Adolescent Psychiatry and Psychology, Clínic Institute of Neurosciences, Hospital Clínic de Barcelona, Barcelona, Spain; 3 Astrid Lindgren Children’s Hospital, Karolinska University Hospital, Stockholm, Sweden; 4 Department of Psychiatry, University of North Carolina at Chapel Hill, Chapel Hill, North Carolina, United States of America; 5 Department of Nutrition, University of North Carolina at Chapel Hill, Chapel Hill, North Carolina, United States of America; 6 Department of Psychological and Brain Sciences, Indiana University, Bloomington, Indiana, United States of America; 7 Medical Affairs, Shire Sweden AB, a Takeda Company, Stockholm, Sweden; 8 Global Medical Affairs, Shire International GmbH, a Takeda Company, Zug, Switzerland; 9 School of Medical Sciences, Örebro University, Örebro, Sweden; University of Oviedo, SPAIN

## Abstract

**Background:**

Individuals with ADHD are at increased risk for poor occupational outcomes. Educational attainment and psychiatric comorbidity may be important contributing factors for these outcomes, but the role of these factors is not well characterized. This study aimed to investigate the associations between ADHD and occupational outcomes, and to examine the influence of educational attainment, comorbid developmental disorders and intellectual disability on these associations.

**Methods:**

We linked the Swedish population graduating from compulsory school 1998–2008 (N = 1.2 millions) to population-wide register-based data on clinical psychiatric diagnoses and medications, objective annual measures of educational, and occupational outcomes. Individuals were followed for between 6 to 16 years after graduation.

**Results:**

Individuals with ADHD had annually on average 17 percent lower income, ratio = 0.83 (95% CI 0.83–0.84), 12.19 (11.89–12.49) more days of unemployment, and a higher likelihood of receiving disability pension, odds-ratio = 19.0 (18.4–19.6), compared to controls. Comorbid diagnoses of intellectual disability and developmental disorder explained most of the association between ADHD and disability pension, while lifetime educational attainment partially explained associations between ADHD and all occupational outcomes. Analyses of occupational trajectories found that income was lower and unemployment elevated relative to controls with the same educational attainment. Higher educational attainment correlated with higher income similarly among individuals with ADHD and controls after accounting for individual background factors.

**Conclusions:**

The occupational burden associated with ADHD is substantial. Comorbid developmental disorders, intellectual disability and educational difficulties (e.g., failing grades) from childhood to adulthood are important factors to consider when designing interventions to improve occupational outcomes in individuals with ADHD.

## Introduction

Attention-deficit/hyperactivity disorder (ADHD) is a prevalent, childhood-onset neurodevelopmental disorder affecting around 5 percent of children [1], and 2.8 percent of adults worldwide [2]. ADHD symptoms may be linked to occupational problems through difficulties in focusing on the current task, planning and organizing tasks, and handling workplace relationships with colleagues [3, 4]. ADHD incurs substantial productivity losses both for adults, and families with affected children [5–7]. The long-term relations between ADHD and occupational outcomes are therefore important to study, for the affected individuals, intervention design, and public health policy.

Studies based on ADHD symptoms have found negative associations with income, and positive associations with welfare dependence, work disability, and unemployment [8–11]. Research based on clinically diagnosed ADHD have identified similar associations [12–16], including a higher likelihood of receiving public assistance (e.g., welfare payments) (odds ratio [OR] = 8.7) [14], and unemployment (OR = 1.39) [12], lower income (-33%) [17], and lower income when employed [18], than individuals without ADHD. However, several findings have been inconsistent. For example, inattentive, but not hyperactive symptoms of ADHD associate negatively with income [11], and several studies have found significant differences between individuals with ADHD and controls for unemployment, but not for income [15, 19]. As most studies have been based on self-reported measures of ADHD and occupational outcomes in clinically based, small samples (typically N<600 individuals with ADHD), problems inherent in such data (e.g., attrition, recall bias) may contribute to the observed inconsistencies in results [20]. It is therefore important to examine these relations using objective measures of ADHD and occupational outcomes. Factors that may be important in explaining the relation between ADHD and occupational outcomes is that ADHD presents with high rates of co-occurring intellectual and psychiatric problems [21, 22], and lower levels of educational attainment (EA), including secondary (e.g., high school) [8, 23, 24], and tertiary (e.g., university) [25] levels. Available studies have found that psychiatric comorbidity was indicative of work disability [8], and a negative correlation between lifetime depression and work participation among individuals with ADHD [26]. An important limitation in these studies has been the lack of a control group without ADHD, which makes it difficult to assess whether the influence of these comorbidities on occupational outcomes are unique to ADHD. While the association between ADHD and lower EA could explain subsequent occupational outcomes [20], a caveat is that ADHD is also negatively correlated with EA at a genetic level [27, 28]. If individual genetic predispositions explain the positive correlations between higher EA and better occupational outcomes, rather than the education itself, the importance of EA for occupational outcomes in ADHD may be more limited than expectations based on observed correlations between EA and occupational outcomes in the general population. Research using genetically sensitive designs has found that EA played a minor role for occupational outcomes when comparing siblings discordant for self-reported ADHD [17], while another study found that EA strongly mediated the association between a polygenic score for ADHD and income (14–58 percent) [29]. As neither of these studies examined these relations longitudinally (i.e., over time), the extent to which the influence of EA on occupational trajectories differs between individuals with and without ADHD is unknown.

Overall, the current knowledge on ADHD and occupational outcomes is based on cross-sectional, often self-reported measures, of ADHD and occupational outcomes. We advance this field using longitudinal, individually linked, register-based measures of occupational outcomes covering the entire Swedish population of compulsory school graduates 1998–2008. These data comprised 1.2 million individuals, including 28.9 thousand diagnosed with ADHD, which contributed 12.4 million yearly observations of occupational outcomes among controls and 285 thousand among individuals with ADHD to our analyses. We addressed three questions regarding ADHD and occupational outcomes. First, we estimated the association between ADHD and occupational outcomes. Second, we explored the role of developmental disorder and intellectual disability, and lifetime EA, in explaining these associations. Third, we examined the influence of EA on occupational trajectories before and after an educational completion while accounting for unobserved individual characteristics, including genetic factors.

## Methods

### Data sources

We extracted data on all 1,221,453 individuals graduating from Swedish compulsory school between 1998 and 2008 from the National School Register (NSR). With few exceptions, these individuals were born 1982–1992 as compulsory school graduation in Sweden usually takes place the year individuals turn 16. These individuals were then linked to demographic information in the Total Population Register (TPR), and annual measures of occupational outcomes and EA from 1998 to 2013 in the Longitudinal Integration Database for Health Insurance and Labor Market Studies (LISA). LISA covers the entire Swedish population aged 16–65. The National Patient Register (NPR) and the Prescribed Drug Register (PDR) were used to gain information on medical diagnoses and dispensed medication respectively. The NPR includes principal and secondary in/outpatient diagnoses (outpatient data since 2001) coded using the International Classification of Diseases (ICD). The PDR includes information on the Anatomical Therapeutic Chemical classification system (ATC) for each medication dispensed since July 2005. The Regional Ethics Committee in Stockholm, Sweden, approved this study (diary number 2013/862-3115). Research using anonymized Swedish register data does not require informed consent from participants.

### Definitions of outcomes, exposures, and covariates

#### Occupational outcomes

Three annual occupational measures were included from LISA: *Income* was indexed by disposable income (100s of Swedish crowns) equal to the difference between income from assets (e.g., wage, social benefits) and liabilities (e.g., interest payments, taxes). We inflated income to 2019 prices, and converted to euro (average exchange rate 2019, 10.59 SEK per euro) [30]. *Unemployment* is measured in days an individual was registered as seeking employment through the Swedish Public Employment Service (0 to 365 days). *Disability pension* is granted to individuals with impaired work capacity due to illness or injury and amounts to 65 percent of lost income.

#### ADHD

In accordance with prior research using Swedish register data, ADHD was coded as a binary variable, defined as a lifetime (i.e., observed at any time point during follow-up) principal diagnosis of ADHD (ICD10: F90) and/or a lifetime dispensation of medication for ADHD, including amphetamines (ATC: N06BA01-02), methylphenidate (N06BA04), or atomoxetine (N06BA09) [31]. This definition of ADHD has been validated in a prior publication from our group, and shows a very strong, positive correlation with ADHD symptoms (Cohen’s *d* = 1.74) [32].

#### Intellectual disability and developmental disorder

Using the NPR, we included lifetime, principal or secondary diagnoses of developmental disorder (ICD10: F80-89, e.g., speech and language disorders, autism spectrum disorders) or intellectual disability (ICD10: F70-79), henceforth abbreviated DD/ID. While a broader definition of psychiatric comorbidity may be considered, including other psychiatric illnesses (e.g., depression) may be problematic as they may be influenced by both ADHD and occupational outcomes (e.g., income level) [33], thereby biasing the associations between ADHD and occupational outcomes.

#### Educational attainment

EA was indexed by year of completion according to three levels: 9 years of compulsory education (reference), a secondary education of 3 years, or a tertiary education of 2 years or more. Compulsory school graduation usually takes place at age 16, and graduation from a secondary education 3 years later, but can for various reasons happen later (e.g., dropping out and completing it later). For individuals with less than 30 percent missing observations of EA, we imputed missing EA using the individual’s available observations which affected 1.5% of individuals with ADHD, and 0.4% of controls. Individuals with higher rates of missingness were excluded (2% ADHD, 1.9% controls). Details on imputations and exclusions are available in S1 File.

#### Covariate

Indicators of individuals’ sex (1 when female, 0 otherwise) and immigrant status (1 when immigrant, 0 otherwise) were included from the TPR. Year of compulsory school graduation (1998–2008, 11 levels) was available in the NSR. LISA provided annual information on whether an individual was receiving study benefits (1 when true, 0 otherwise). Study benefits are government transfers to individuals who are enrolled in an educational program, and consist of a combination of an optional loan and an allowance.

After exclusions (see S1 File for details) 1,196,744 individuals remained (98 percent of the source population) which contributed 12,686,016 annual observations for analysis.

### Analyses

Means (standard deviations) and frequencies (percent) in the first and last year of follow-up were reported for continuous (income and unemployment), and categorical (disability pension) outcomes respectively.

#### Modeling strategy

As each of our occupational outcome measures differed in terms of numerical range, we begin by defining three covariate sets used across analyses, then outline the shared aspects of the models for each outcome, and finally how these were adapted to address our three aims.

*Covariate sets*. To account for cohort effects (e.g., differing length of follow-up) and yearly trends in the outcomes, we defined a *cohort* set including index year of observation (i.e., year 0–15 after compulsory school graduation) and year of compulsory school graduation (1998–2008). The *demographic* set included cohort covariates as above, plus sex and immigrant status. When analyzing income and unemployment we also included a *mediation* covariate set (defined below for each of our three outcomes) to account for the degree of labor market absence. Thus, when using the mediation covariates, the analyses targeted income and unemployment among those that were in employment. Additional covariates that varied across outcomes are defined below.

*Income*. In our main analyses of income we excluded negative observations (2.3% ADHD, 1.7% controls), applied a logarithmic transformation, and analyzed this outcome using linear regression models. The logarithmic transformation was applied to reduce skewness of the income distribution, and for comparative purposes as estimated associations are easily converted to ratios/percentages [34]. We reported the exponentiated coefficients from these models, which equal the estimated income ratio (IR) due to a unit increase in the exposure. Analyses of the untransformed income variable were provided as supplementary analyses. The *mediation* covariate set included days unemployed, indicators of study benefits, and disability pension respectively, as time-varying covariates.

*Unemployment*. We estimated the difference in unemployment days using linear regression. The *mediation* covariate set included indicators of disability pension and study benefits respectively, to account for the degree of labor market absence.

*Disability pension*. We estimated the risk of disability pension (present versus not present) using logistic regression, and reported odds-ratios (ORs). We also reported risk differences (RDs), defined as the difference in the probability of disability pension among individuals with ADHD compared to controls. An indicator was included in the models to account for an age restriction implemented in 2003, 1 if the year was 2003 or later and the individual was aged 19 years or older, 0 otherwise.

#### Association between ADHD and occupational outcomes

First, we estimated the average differences in occupational outcomes between individuals with and without ADHD from compulsory school graduation to end of follow-up, adjusting for the cohort covariate set. Second, we adjusted for both the demographic and mediation covariate sets to see the extent to which the associations were explained by that individuals with ADHD may be more absent from the labor market where income may reflect various government transfers (e.g., study benefits).

#### Influence of lifetime DD/ID and EA on the association between ADHD and occupational outcomes

To address this aim, we extended the models outlined in the paragraph above and adjusted the association between ADHD and occupational outcomes for lifetime DD/ID and lifetime EA (secondary and tertiary levels) in separate analyses.

#### Influence of EA on occupational trajectories in ADHD

As the preceding analysis of lifetime EA does not give any indication of the extent to which EA itself alters occupational trajectories, we estimated the associations between ADHD and occupational outcomes on an annual basis before and after completing a secondary or tertiary education.

*Residual plots*. First, to clarify the temporal patterns that these models analyze, we regressed the cohort covariate set on income and unemployment, and plotted means and 95 percent confidence intervals of residual variation (i.e., the variation not explained by the cohort covariates). We calculated these statistics over index year of observation, lifetime EA level, and presence of ADHD. As the relationship between EA and occupational outcomes is dependent on the length of follow-up after an educational completion, we added estimates of the average graduation year in ADHD and controls, adjusted for year of compulsory school graduation, in these plots.

*Model outline*. Second, we modeled the influence of EA on occupational trajectories in ADHD by creating indicators of secondary and tertiary EA as time-varying exposures (0 before an EA, 1 after), and their interactions with ADHD status and index year after educational completion. We thereby compared the period before with the period after an educational completion in terms of a level effect (i.e., permanent mean difference) and a linear annual trend (i.e., annual difference), with and without the presence of ADHD. Index year of observation was included as a population fixed effect (16 levels), and we applied a within individual (i.e., fixed effects) approach [35] to account for unobserved individual characteristics that may correlate with EA and ADHD, Unobserved individual characteristics include factors constant over time, such as genetic make-up, school of graduation, and others. Time varying covariates where retained in these models. As we have defined ADHD as a diagnosis/medication dispensations at any time during follow-up, it is only possible to estimate how ADHD modifies the main effects of EA through the interaction terms specified above. A lagged value (i.e., the outcome at *t* regressed on the outcome at time *t-1*) was included as a covariate in the model for income to account for serial correlation. For instance, individuals in employment will gain experience that affects income and the lags can thus be thought of as proxies of this unobserved variable.

*Model illustration*. To illustrate the estimated associations, we predicted occupational trajectories from the fitted models under two different scenarios, one where everyone in the population was affected by ADHD to one where none was. Each prediction was done 1000 times, drawing individuals at random with replacement. We computed ratios of occupational outcomes by year after compulsory school graduation and lifetime EA, and 95 percent confidence intervals based on quantiles. See S1 Methods for details.

*Sensitivity analyses*. As within individual models including lagged outcomes may be susceptible to bias that may overestimate the effects of the exposures [36], we performed sensitivity analyses by estimating occupational trajectories in a within-model without lags and a between model with lags.

Analyses were performed in R version 3.6.1 using package lfe [37] for continuous and bife [38] for dichotomous. Routines in bife were used to calculate RDs. Robust standard errors, accounting for deviations from distributional assumptions (e.g., normally distributed residuals), heteroscedasticity, and dependencies between row of data, clustered on individuals were used in all analyses for calculating confidence intervals.

## Results

Of the Swedish population graduating compulsory school between 1998 and 2008, 2.4 percent were affected by ADHD. Among individuals with ADHD, the proportion of females (40.9% in ADHD vs 48.9% among controls) and immigrants (6.3% vs 8.1%) were lower compared to controls. Higher rates of mortality (1.0% versus 0.2%), developmental disorder (18.6% versus 0.7%) or intellectual disability (2.5% versus 0.1%), and lower lifetime EA were observed among individuals with ADHD compared to controls. For instance, rates of lifetime tertiary EA in the oldest cohort that graduated in 1998 was 13.9 percent in the ADHD group compared to 44.5 percent among controls (Table 1). Within the ADHD group, a minority (17%) had never been dispensed an ADHD medication. The main difference in background characteristics between individuals with and without a dispensation of ADHD medication was a higher proportion of females in the group with a dispensation, 42.6% compared to 33.1% among those without a dispensation. Other differences were small (<1%) (S1 Appendix).

**Table 1:** Descriptive statistics.

		**ADHD**			**Controls**		**P-value**^**a**^
Individuals		28,914 (2.4)			1,167,830 (97.6)		<0.001
Female		11,839 (40.9)			570,840 (48.9)		<0.001
Immigrant		1,824 (6.3)			94,966 (8.1)		<0.001
At least 1 missing observation of EA		1,422 (4.9)			65,509 (5.6)		<0.001
Deceased		291 (1.0)			1,814 (0.2)		<0.001
Diagnosis of developmental disorder		5,379 (18.6)			8,705 (0.7)		<0.001
Diagnosis of intellectual disability		715 (2.5)			1,570 (0.1)		<0.001
Lifetime educational attainment and prevalence of ADHD by year of compulsory school graduation (%)							
**Graduation year**	**ADHD**	**Compulsory**	**Secondary**	**Tertiary**	**Compulsory**	**Secondary**	**Tertiary**
**1998**	1.7	37.3	48.8	13.9	8.5	47.0	44.5
**1999**	1.8	35.6	52.3	12.1	7.9	49.5	42.7
**2000**	1.8	35.0	52.8	12.1	7.7	50.5	41.8
**2001**	1.9	35.4	53.2	11.4	7.5	53.0	39.5
**2002**	1.9	35.4	54.7	9.9	7.3	55.3	37.4
**2003**	2.2	35.9	57.1	7.0	7.4	57.9	34.7
**2004**	2.3	39.3	54.8	5.9	7.7	61.5	30.9
**2005**	2.7	39.4	56.5	4.1	8.2	65.4	26.4
**2006**	2.9	39.8	56.7	3.4	8.6	71.4	20.0
**2007**	3.1	40.8	57.8	1.4	8.9	79.1	12.1
**2008**	3.6	42.3	57.3	0.4	9.1	87.4	3.5

N (%) unless specified.

^a^ P-value calculated based on Pearson’s chi-squared test of counts in each variable (e.g., female sex) over ADHD status.

### Association between ADHD and occupational outcomes

Crude differences in occupational outcomes between individuals with and without ADHD were present both in the first (t_0_) and last year of follow-up (t_15_). Nominal income among individuals with ADHD was t_0_ = €857 and t_15_ = €15,816 and among controls t_0_ = €935 and t_15_ = €23,098. Unemployment was t_0_ = 0.2 and t_15_ = 28.9 days for individuals with ADHD, and t_0_ = 0.0 and t_15_ = 11.7 days for controls. Presence of disability pension (percent) was found for t_0_ = 160 (0.6%) and t_15_ = 188 (11.7%) individuals with ADHD, and for t_0_ = 882 (0.1%) and t_15_ = 787 (0.9%) of controls. When adjusting for demographic and mediation covariates, individuals with ADHD had on average during follow-up, 17 percent lower income (income ratio [IR] = 0.83), 11.59 more days per year of unemployment, and a 18.99 times higher odds, risk difference (RD) = +11.0%, of disability pension, compared to individuals without ADHD (Table 2). Estimates of nominal income differences are available in S1 Table.

**Table 2:** Associations between ADHD and occupational outcomes, and the influence of comorbid intellectual disability/developmental disorders and lifetime educational attainment.

	Observed occupational outcome				Modeled			
	Year after compulsory school graduation				Covariate sets adjusted for			
	0	15	0	15	Cohort	Demographic and mediation	Demographic, mediation, and comorbid ID/DD	Demographic, mediation, and lifetime EA
	**ADHD**		**Controls**		**ADHD**			
	**Mean EURO (SD)**				**Income ratio, exp(β)**			
**Income**	857.0 (1372.0)	15,816.3 (8,835.5)	935.0 (3,035.7)	23,097.8 (15,474.7)	0.81 (0.80, 0.81)	0.83 (0.83, 0.84)	0.86 (0.86, 0.87)	0.86 (0.86, 0.87)
	**Mean days (SD)**				**Unemployment days, β**			
**Unemployment**	0.2 (3.5)	28.9 (70.9)	0.0 (1.7)	11.7 (45.9)	11.84 (11.55, 12.14)	12.19 (11.89, 12.49)	11.59 (11.28, 11.90)	8.33 (8.04, 8.63)
	**Frequency (%)**				**Odds-ratio, exp(β); % risk-difference**			
**Disability pension**	160 (0.6)	188 (11.7)	882 (0.1)	787 (0.9)	18.8; 10.9 (18.2, 19.4)	19.0; 11.0 (18.4, 19.6)	5.68; 3.0 (5.36, 6.02)	8.63; 4.7 (8.30, 8.97)

t: Year (0–15) after graduation. *Cohort adjustment* include year of compulsory school graduation (11 levels) and year of observation (16 levels) as fixed-effects. *Demographic adjustment* include cohort covariates, sex, and immigrant status. *Mediation* covariates varied by outcome as follows: Income included days unemployed, presence of study benefits and disability pension respectively. Unemployment included disability pension and presence of study benefits. Disability pension included an indicator of being at least 19 years of age in 2003 or later due to an eligibility requirement implemented this year. *Comorbidity* includes indicators for a lifetime diagnosis of developmental disorder or intellectual disability. *Lifetime EA* includes indicators of lifetime secondary and tertiary educational attainment.

### Influence of lifetime ID/DD and EA on the association between ADHD and occupational outcomes

Adjusting for lifetime comorbid DD/ID decreased the income difference to -14 percent (IR = 0.86), and more than halved the odds-ratio of disability pension to 5.68, RD = +3.0%, while unemployment was marginally affected. Adjusting for lifetime secondary and tertiary EA influenced the income ratio, and the risk of disability pension similar to adjusting for comorbid ID/DD, but also attenuated the unemployment difference to 8.33 days (Table 2).

### Influence of EA on occupational trajectories in ADHD

**Fig 1:**
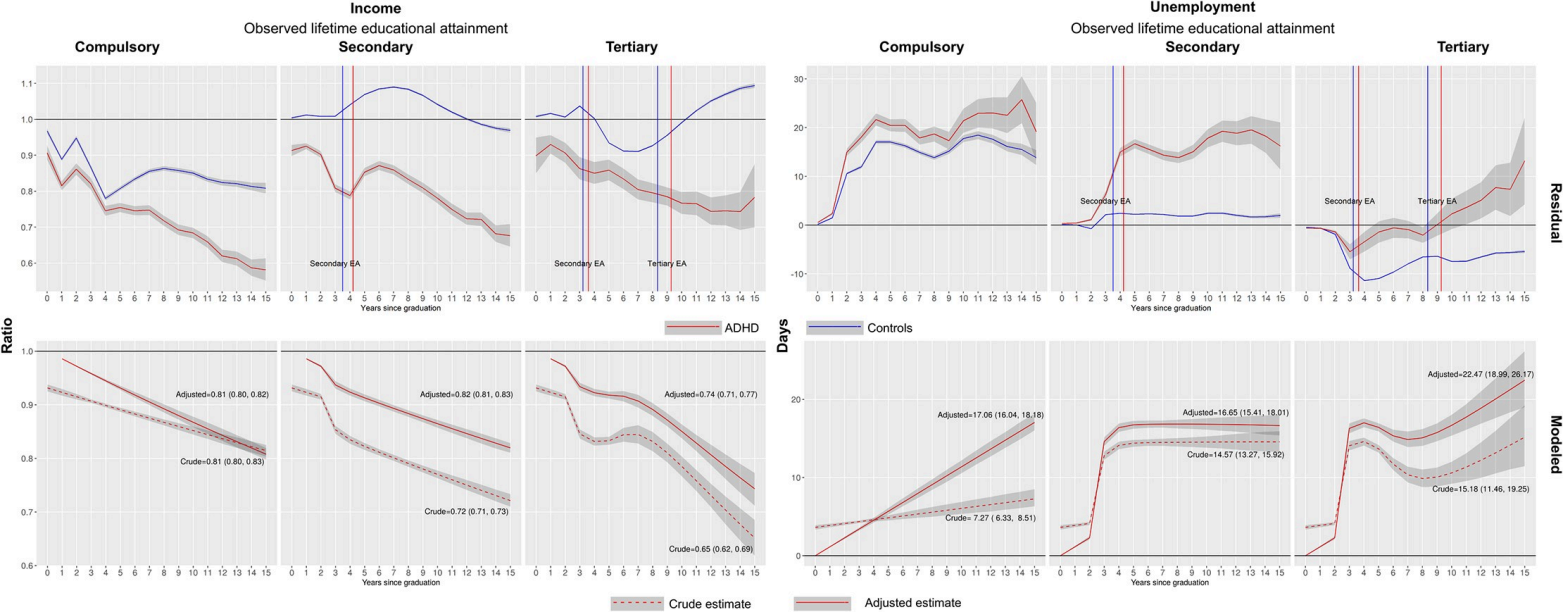
Influence of ADHD on occupational trajectories by highest observed educational attainment. **Residual panel:** Influences from cohort (i.e., year of graduation from compulsory school) and index year of observation have been removed through regression before calculating means and confidence intervals. The observations are thus relative to the population trend in each outcome, indicated by the horizontal lines. Vertical lines in plots for secondary and tertiary educational attainment indicate the average year after compulsory school graduation for each educational level among individuals with (dotted) and without (solid) ADHD respectively. **Modeled panel:** Estimates of income and unemployment relative to controls (horizontal lines). The dashed line represent crude estimates (e.g., the income ratio of ADHD to controls) while the full line represent estimates from the adjusted model. For details on the model, see main text. Texts inside plots are the estimated income ratios and unemployment days at end of follow-up (i.e., year 15). The models for the adjusted income ratios include a lag, thus these curves drop the first observation and begin at year one of follow-up.

The *residual* panels of Fig 1 shows that, within each level of lifetime EA (compulsory, secondary or tertiary), income declined steadily and unemployment was persistently elevated during follow-up among individuals with ADHD (red line) relative to individuals without (blue line). Occupational differences between individuals with and without ADHD were also apparent prior to these educational attainments as indicated by the vertical red (ADHD), and blue (controls) lines. The distance between these lines indicated that individuals with ADHD graduated from the secondary/tertiary educations about one year later than controls (estimates available in S2 Table).

The modeled differences between individuals with and without ADHD are plotted in the lower panels (“modeled”) of Fig 1 (estimates available in S3 Table). The dashed lines represent crude associations (i.e., a quantification of the annual differences associated with ADHD in the residual panels) adjusted for the cohort covariates, and the solid line the within individual associations adjusted for index year of observation, mediation covariates, and in the case of income a lagged value of income. At the end of follow-up (year 15 after compulsory school graduation), income among individuals with ADHD compared to individuals without, were estimated to 19 percent lower (IR = 0.81 [0.80, 0.82]) for lifetime compulsory EA, 18 percent lower (IR = 0.82 [0.81, 0.83]) for secondary lifetime EA, and 26 percent lower (IR = 0.74 [0.71, 0.77]) for tertiary EA. The corresponding unemployment differences were estimated to 17.06 (16.04, 18.18) days higher for compulsory EA, 16.65 (15.41, 18.01) days higher for secondary EA, and 22.47 (18.99, 26.17) days higher for tertiary EA.

**Fig 2:**
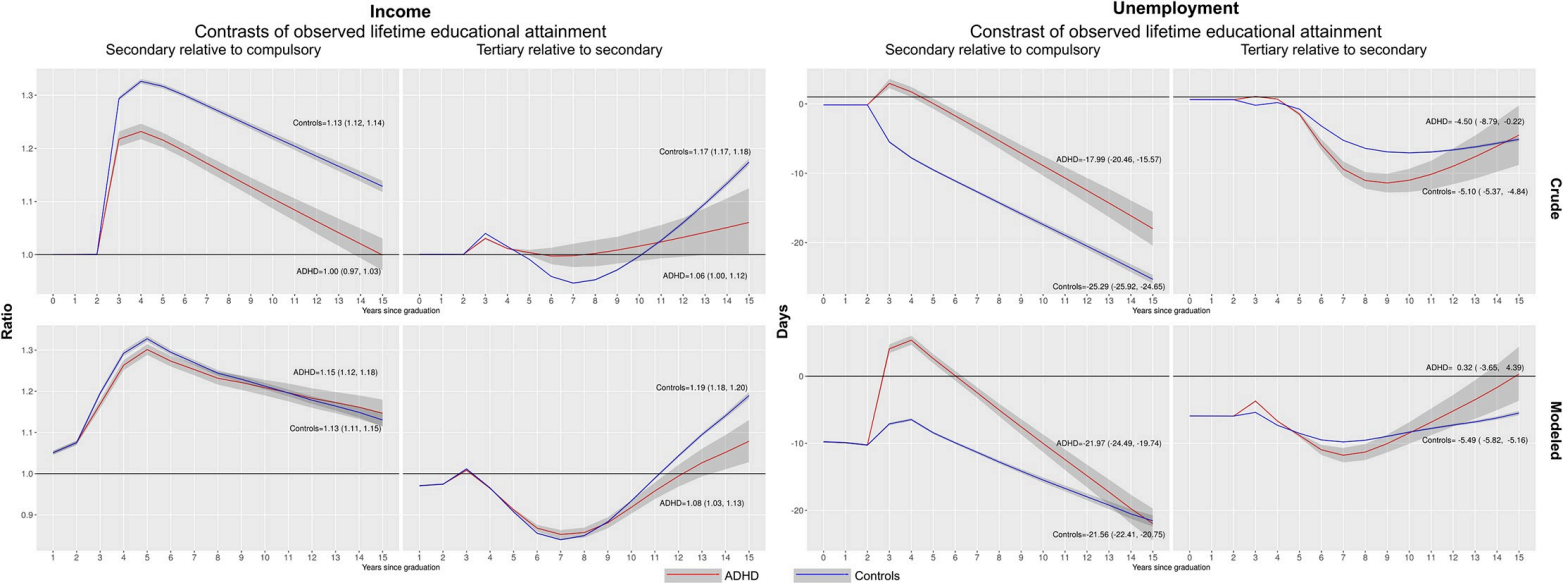
Influence of educational attainment on occupational trajectories in ADHD. Income ratios and unemployment differences of individuals with a secondary, and tertiary lifetime educational attainment relative to individuals with compulsory and secondary lifetime educational attainment, among individuals with (red line) and without (blue line) ADHD. Texts inside plots are the estimated income ratios and unemployment days at end of follow-up (i.e., year 15). **Crude panel:** Income and unemployment estimates adjusted for influences from cohort (i.e., year of gradation from compulsory school), and index year of follow-up. **Adjusted panel:** Income and unemployment estimates adjusted for influences from the mediation covariate set. For details, see main text. The models for the adjusted income ratios include a lagged value of income, and these curves thus drop the first observation and begin at year one of follow-up.

While these results show lower income and higher unemployment among individuals with ADHD relative to controls, the extent to which EA influences occupational trajectories within these groups is less clear. Fig 2 plots income and unemployment differences where the group with higher EA are compared to those with a lower EA (i.e., secondary to compulsory EA, and tertiary to secondary EA) among individuals with ADHD and controls respectively. Crude income ratios of secondary to compulsory EA, implicated a higher income in the group with a secondary EA, but this ratio was lower among individuals with ADHD compared to controls (Fig 2, upper left panel). When adjusting for individual background factors these differences were no longer present as the confidence-intervals overlapped entirely (Fig 2, lower left panel). The income ratio for a tertiary relative to a secondary EA displayed a different pattern where the income ratio was higher among individuals with ADHD compared to individuals without during part of the follow-up. At the end of follow-up the income ratio was higher among controls than among individuals with ADHD, but this difference was less pronounced after adjusting for individual background factors. Unemployment differences, comparing secondary to compulsory EA showed a clear peak around year 3 of follow-up, but then steadily declined and was similar to the unemployment difference among those with a secondary EA at end of follow-up. In contrast, tertiary compared to secondary EA, indicated lower unemployment among those with a tertiary EA, with a minor difference between individuals with and without ADHD.

The sensitivity analyses of income trajectories concurred with the overall patterns, but suggested smaller differences between individuals with and without ADHD for the models including a lagged outcome only, while models including individual fixed-effects only suggested stronger differences (S1 Fig).

## Discussion

Using virtually the entire Swedish population of 1,196,744 individuals graduating compulsory school 1998 to 2008 with up to 16 years of follow-up, this study identified several important associations between ADHD and occupational outcomes, and their interplay with EA and comorbid DD/ID. The association between ADHD and lower income, higher unemployment, and a higher risk of disability pension, was not entirely explained by comorbid DD/ID or EA. Lifetime EA explained part of the associations between ADHD, income and unemployment respectively, while comorbid DD/ID were important determinants of the elevated risk of disability pension in ADHD. Notably, when accounting for unobserved individual characteristics, the longitudinal effects of ADHD on income and unemployment within the same EA, were roughly similar in magnitude relative to controls in all educational strata (compulsory, secondary, and tertiary). This suggests that important differences in occupational outcomes between individuals with and without ADHD are driven by other factors than EA. These results are of importance for policy makers and other stakeholders, both in terms of awareness of occupational functioning amongst individuals with ADHD, and regarding expectations on education as a determinant of future occupational performance among individuals with ADHD.

Our findings regarding the crude associations between ADHD and occupational outcomes are consistent with previous research using similar definitions of ADHD, and research exploring associations with other psychiatric conditions and behavioral problems [10, 18, 39–41]. Our estimates of crude income differences between individuals with ADHD and controls at the end of follow-up (around age 30) ranged between -19 to -35 percent across EAs (Fig 1). In a US-based sample of similar age, self-reported ADHD associated with 33 percent lower earnings [17]. A meta-analysis of ADHD and long-term outcomes reported an increased odds (OR = 1.97 [1.01, 3.85]) of ever being unemployed for individuals with ADHD [25]. Consistent with a Norwegian study on adults clinically diagnosed with ADHD which found that the number of comorbid disorders were predictive of long-term work disability [8], we found that a comorbid diagnosis of DD/ID explained the substantially elevated risk of disability pension in ADHD.

Three important patterns emerged regarding the role of EA for the association between ADHD and occupational outcomes. First, we found that adjusting for lifetime EA decreased the income difference between individuals with and without ADHD, which is consistent with a recent molecular genetic study reporting that the association between the polygenic risk of ADHD and income was mediated by EA by 14–58 percent [29], and a number of quantitative [42] and molecular [43] genetic studies demonstrating negative genetic correlations between ADHD and EA. Second, individuals with and without ADHD differ from each other in terms of occupational outcomes prior to educational completions (residual panels, Fig 1). When we accounted for these differences, and explored the longitudinal relations between EA and occupational outcomes in ADHD, we found that ADHD influenced income and unemployment relative to controls similarly within each level of lifetime EA. Third, when analyzing the relative influence of EA (e.g., secondary compared to compulsory EA) we found that the income trajectories among individuals with ADHD were very similar to those in controls when accounting for unobserved individual characteristics (Fig 2). Still, the corresponding unemployment trajectories differed in this aspect, as ADHD was associated with elevated unemployment when comparing secondary with compulsory EA. An explanation of these patterns needs to take into account both that ADHD associates with poor occupational outcomes compared with controls regardless of EA (Fig 1), and that the relative influence of higher compared with lower EA is similar within the ADHD group and controls for income, but different for unemployment (Fig 2). Prior research has identified an association between ADHD symptoms and lower wages for employed individuals [10], and a study from Sweden found that a mismatch between educational level and type of employment was associated with a 20 percent reduction in income compared with when successful matching had occurred [44]. Potentially, the weaker influence of secondary than compulsory EA on unemployment among individuals with ADHD could mean that individuals with ADHD are less successful in finding and keeping employment that matches their educational level after graduation (e.g., having a carpentry education and working as a desk clerk/receptionist) compared to controls. That ADHD associates with more frequent unemployment has been observed in several studies [12, 25]. Our results highlight that this association is not entirely explained by lower EA among individuals with ADHD.

Finally, these patterns challenge the idea that post-compulsory EA itself explains occupational differences between individuals with and without ADHD. Factors in ADHD that correlate with occupational outcomes, such as genetic predispositions [29] and developmental patterns in childhood/early adolescence [10, 11], are likely of continued importance for the role of EA in the association between ADHD and adult occupational outcomes. Consistent with prior research showing less progression from community/junior college to four-year college/graduate school in the US among individuals with ADHD compared to controls [45], we find that ADHD is associated with delayed secondary/tertiary graduation compared to controls (Fig 1 and S1 Table). This observation may indicate that students with ADHD allocate time spent in occupational, educational, and recreational activities differently than controls, leading to later problems finding employment. Future research should devote attention to determinants of occupational activity in adolescence/early adulthood (e.g., apprenticeships, part-time jobs during studies), whether individuals with ADHD differs in this respect, and how such activities may correlate with later occupational outcomes.

### Strengths and limitations

Our study strengthens the existing knowledge on ADHD and occupational outcomes, and also extends the knowledge base by proposing a longitudinal role of EA for occupational outcomes in ADHD. Contrary to many prior studies on this topic, we had access to a large sample of individuals with ADHD in combination with objective measures of both occupational outcomes and EA. Nevertheless, we would like to highlight several important limitations.

First, the generalizability of our results may be sensitive to the presence of pharmacologically treated ADHD. In our sample, 83 percent of individuals with ADHD had at some point been dispensed an ADHD medication. While the comparison group here is small, we find that individuals with ADHD and a dispensed ADHD medication had slightly lower income (IR = 0.98 [0.96, 0.99]), lower unemployment (-2.86 [-3.68, -2.04] days), and higher risk of receiving disability pension (OR = 1.18 [1.08, 1.28]) compared with those without such a dispensation (S1 Appendix). A more detailed analysis, considering factors such as treatment duration, confounding by indication (i.e., that treatment may indicate poor occupational outcomes), of the influence of ADHD medication on occupational outcomes in ADHD is warranted. But as the Swedish prescribed drug register is only available from 2005 onwards, we are limited insofar as the length of follow-up is systematically correlated with age in our sample. We acknowledge that our results may be more valid for pharmacologically treated ADHD, and highlight the need for future research to investigate this topic.

Second, the rates of clinically diagnosed ADHD in our source population increased from 1.7 percent among 1998 compulsory school graduates to 3.6 percent among those graduating in 2008 (Table 1). The clinical prevalence of ADHD in our source population is thus lower than more recent estimates in Sweden. Potentially, the registers capture cases with more severe presentations of ADHD during our study period, which may have inflated our estimates of the association between ADHD and occupational outcomes. In addition, since the follow-up on tertiary EA is mainly based on observations from older individuals, this may also have attenuated the influence of tertiary EA on the association between ADHD and occupational outcomes. Our within individual analyses account for such factors to some extent, but with an increasing diagnostic prevalence of ADHD, these unobserved individual factors may correlate differently with EA than in the current study population.

Third, as our unemployment measure only captures individuals actively seeking employment through the official employment agency, correlations between ADHD and other means of employment seeking may bias our estimates.

Fourth, regarding generalizability of our results, access to education is facilitated by tuition-free universities and state-provided financing for students in Sweden. Such policies, and labor market structures (e.g., unemployment benefits), are important variables to consider when comparing our findings to that of other countries.

## Conclusion

ADHD is associated with poor occupational outcomes, and differences in psychiatric comorbidity and EA partly explain these associations. Although higher EA is associated with higher income and lower unemployment among individuals with ADHD over time, disparities between individuals with and without ADHD are persistent, and exist regardless of EA. When designing policies to improve occupational outcomes for individuals with ADHD, educational interventions should likely be weighed against alternatives (e.g., job training) while considering the impact of factors such as age, comorbid intellectual disability and developmental disorders.

## Supporting information

S1 File Outline of study population, imputations, and descriptive statistics by ADHD medication status. (DOCX)

S1 Method Within individual modeling and predicted effects of ADHD and educational attainment on occupational trajectories. (DOCX)

S1 Appendix Analyses of ADHD medication status and occupational outcomes. (DOCX)

S1 Table Analyses of nominal income. (DOCX)

S2 Table Association between ADHD and graduation year from secondary/tertiary educations. (DOCX)

S3 Table Influence of educational attainment on occupational trajectories in ADHD. (DOCX)

S1 Fig Sensitivity analyses of income trajectories. (DOCX)
