# Supplementary material for: Attention-deficit/hyperactivity disorder and occupational outcomes: The role of educational attainment, comorbid developmental disorders, and intellectual disability
Source: PLoS One. 2021 Mar 17;16(3):e0247724. doi: 10.1371/journal.pone.0247724 (PMC7968636; doi:10.1371/journal.pone.0247724)
Supplement: S1 File — (DOCX) [file pone.0247724.s001.docx]

S1 File: Outline of study population, imputations, and descriptive statistics by ADHD medication status

**Outline of study population and imputations**

The study population was defined as those graduating compulsory school between 1998 and 2008. Thus this population was per definition alive and resident in Sweden at the start of follow-up. The population was followed until 2013 (i.e., 6 to 16 years of follow-up), giving an age range at end of follow-up from 21 (2008 graduates) to 31 (1998 graduates) years. While individuals with more than 30 percent missing values of educational attainment (EA) during follow-up were excluded, we imputed the missing values of EA for individuals with lower rates of missingness. The imputations were performed in the following order:

1. Missing EA in the ﬁrst three years of follow-up (t =0 to t =2) were set to compulsory school (i.e. the reference level) as graduation from secondary school is observed in t =3.
2. For the remaining missing values, we took the closest, temporally preceding, non-missing value of EA. This was applied sequentially for each individual so that non-missing values of EA were carried forward to "ﬁll-in" later missing values.

As a quality control, we checked how EA changed within each individual over time. A few individuals (24 with ADHD, 230 without) decreased their EA at some point in time. These trajectories were “flattened” and replaced with the lower, observed EA.

| Figure 1. Flow diagram of imputations and exclusions in the extracted study population |
| --- |
| 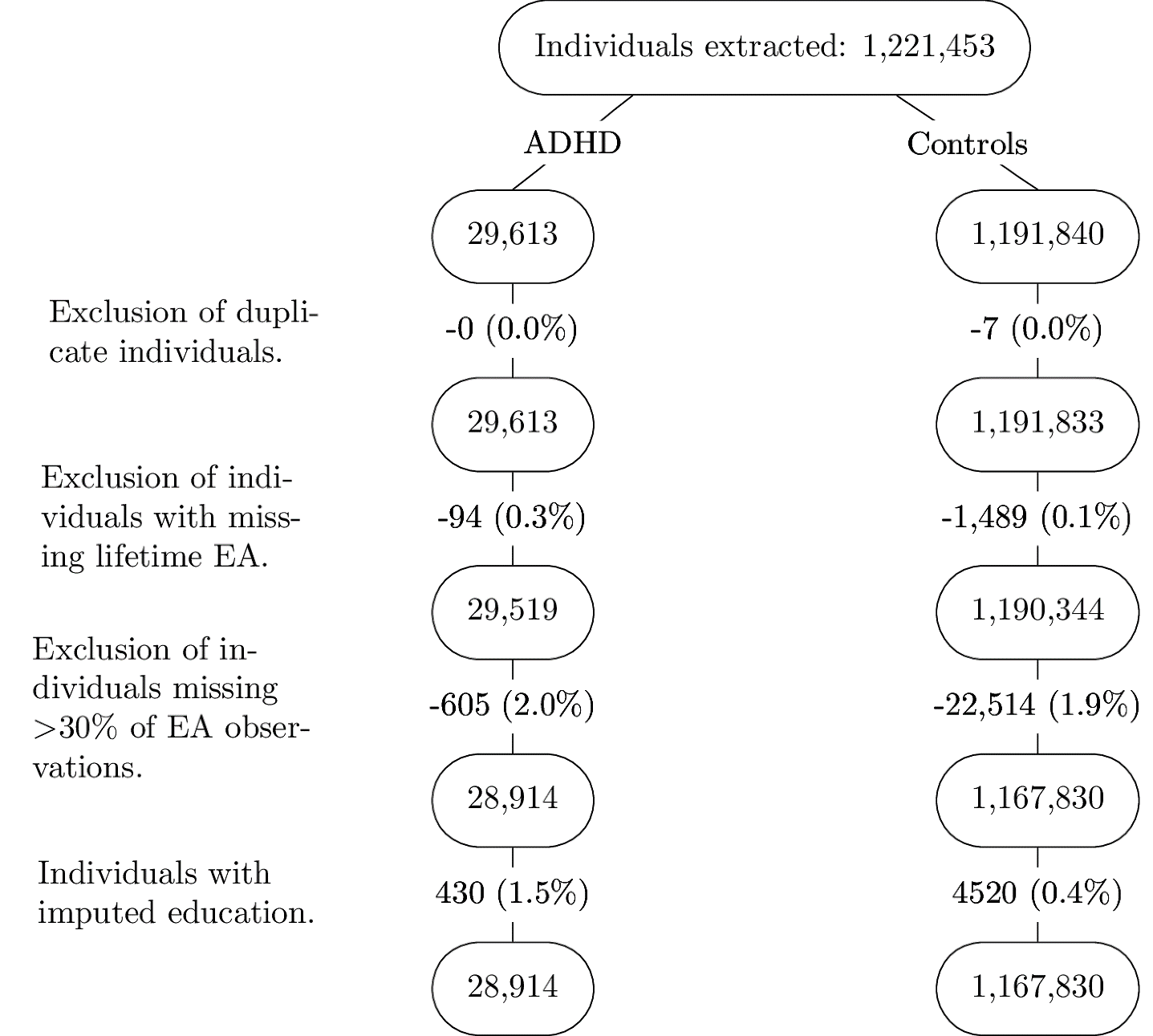 |
| N (%) individuals affected at each step. Negative numbers represent exclusions of individuals and numbers in circles, the study population at each step. |
