# Supplementary material for: Attention-deficit/hyperactivity disorder and occupational outcomes: The role of educational attainment, comorbid developmental disorders, and intellectual disability
Source: PLoS One. 2021 Mar 17;16(3):e0247724. doi: 10.1371/journal.pone.0247724 (PMC7968636; doi:10.1371/journal.pone.0247724)
Supplement: S1 Method — (DOCX) [file pone.0247724.s002.docx]

S1 Method. Within individual modeling and predicted effects of ADHD and educational attainment on occupational trajectories

**Within-individual models**

Within individual, or other observational units with repeated measurements, models are useful in situations where the presence of unobserved variables (e.g, genetics) likely influence the relationships between exposure and outcome. In our case we have repeated observations on individuals with and without lifetime diagnosis of ADHD, along with measures of educational attainment, occupational outcomes and others (e.g., sex). A simplified model of our analysis could be:

$$y_{it}=\alpha_{i}+\beta_{1}ADHD_{i}+\beta_{2}EA_{it}+\varepsilon_{it} (1)$$

Where we suspect that the components of $\alpha_{i}$ correlate with both ADHD, EA, and $y$. The within-individual approach is essentially the same as including an indicator variable for each individual, which is, however, prohibitive due to the large number of individuals. Instead, the same result can be achieved by demeaning (or centering) the data which is automatically performed by the software we used for estimation. Demeaning the data gives:

$y_{it}-\overline{y_{i}}=\alpha_{i}-\overline{\alpha_{i}}+\gamma_{1}(ADHD_{i}-\overline{ADHD_{i}})+\gamma_{2}(EA_{it}-\overline{EA_{i}})+(\varepsilon_{it}-\overline{\varepsilon_{i}})$ (2)

Where the bar above a variable indicates the mean of that variable within an individual. As $\alpha$ is assumed to exert a constant influence on the associations of interest, demeaning the data removes the influence from these factors. Since this also removes ADHD, we interact ADHD with EA to examine how ADHD modifes the associations between EA and $y$:

$y_{it}=\gamma_{1}EA_{it}+\gamma_{2}EA_{it}\times ADHD_{i}+\varepsilon_{it}$ (3)

In this model the coefficients of interest $\gamma_{1}$ (main effect) and $\gamma_{2}$ (effect modification in the presence of ADHD) are not confounded by the presence of unobserved individual factors in estimating the relationship between EA, ADHD, and occupational outcomes.

For a more detailed account, see Gunasekara (2014).

**Model predictions of income and unemployment**

The model for occupational trajectories was estimated 1,000 times where individuals (along with all their observations) were drawn by their unique identifier with replacement. For each estimated model, the outcome was predicted using the estimated coefficients and multiplying with the data which was modified according to the scenarios listed above, e.g., one prediction when ADHD (and interactions with EA variables) was set to 1 for the whole population, and one in which it was set to 0. Subsequently, we calculated the mean predicted effect (income ratio and difference in days unemployed respectively) by year of follow-up ($t=0$ to $t=15$). We used the quantile method to calculate 95 percent confidence intervals, i.e., the 25th and 975th prediction ordered by their nominal value.
