# Supplementary material for: Attention-deficit/hyperactivity disorder and occupational outcomes: The role of educational attainment, comorbid developmental disorders, and intellectual disability
Source: PLoS One. 2021 Mar 17;16(3):e0247724. doi: 10.1371/journal.pone.0247724 (PMC7968636; doi:10.1371/journal.pone.0247724)
Supplement: S1 Appendix — (DOCX) [file pone.0247724.s003.docx]

S1 Appendix. Analyses of ADHD medication status and occupational outcomes

**Descriptive statistics by ADHD medication status**

| Table 1. Descriptive statistics by dispensed ADHD medication status | | | |
| --- | --- | --- | --- |
|  | Treated | Untreated | P-value^a^ |
| Individuals | 23,992 (83) | 4,922 (17) |  |
| Female | 10,210 (42.6) | 1,629 (33.1) | < 0.001 |
| Immigrant | 1,484 (6.2) | 340 (6.9) | 0.062 |
| At least 1 missing observation of EA | 1,178 (4.9) | 244 (5.0) | 0.917 |
| Deceased | 254 (1.1) | 37 (0.8) | 0.059 |
| Diagnosis of developmental disorder | 4,463 (18.6) | 916 (18.6) | >0.999 |
| Diagnosis of intellectual disability | 577 (2.4) | 138 (2.8) | 0.112 |
| N (%). Treatment status determined by a dispensation of a medication for ADHD (ever versus never) in the Swedish Prescribed Drug Register.  ^a^ P-value calculated based on Pearson’s chi-squared test of counts in each variable (e.g., female sex) over ADHD medication status. | | | |

**Table 2. Associations between ADHD medication treatment status and occupational outcomes, and the influence of comorbid intellectual disability/developmental disorders and lifetime educational attainment**

|  | **Observed occupational outcome** | | | | | **Modeled** | | | | |
| --- | --- | --- | --- | --- | --- | --- | --- | --- | --- | --- |
|  | Year after compulsory school graduation | | | | | Covariate sets adjusted for | | | | |
|  | 0 | | 15 | 0 | 15 | Cohort | | Demographic and mediation | Demographic, mediation, and comorbid ID/DD | Demographic, mediation, and lifetime EA |
|  | **Treated** | | | **Untreated** | | **Treated ADHD** | | | | |
|  | **Mean EURO (SD)** | | | | | **Income ratio, exp(β)** | | | | |
| **Income** | 853.7 (1356.2) | 15,817.3 (8,905.0) | | 881.6 (1,459.4) | 16,027.7 (8,482.0) | 0.98  (0.96, 0.99) | 0.97  (0.95, 0.98) | | 0.97  (0.95, 0.98) | 0.97  (0.95, 0.98) |
|  | **Mean days (SD)** | | | | | **Unemployment days, β** | | | | |
| **Unemployment** | 0.2  (3.4) | 28.0 (69.8) | | 0.2  (4.0) | 35.1 (77.9) | -2.86  (-3.68, -2.04) | -1.76  (-2.55, -0.98) | | -1.76  (-2.55, -0.98) | -1.59  (-2.36, -0.82) |
|  | **Frequency (%)** | | | | | **Odds-ratio, exp(β); % risk-difference** | | | | |
| **Disability pension** | 125 (0.5) | 172  (12.2) | | 35  (0.7) | 16  (8.9) | 1.18  (1.08, 1.28) | 1.16  (1.07, 1.26) | | 1.22  (1.12, 1.33) | 1.19  (1.10, 1.30) |
| Treatment status determined by a dispensation of an ADHD medication in the Swedish Prescribed Drug Register.  Treated: Individuals with a lifetime dispensation of ADHD medication. Untreated: Individuals without a lifetime dispensation of ADHD medication.  t: Year (0-15) after graduation. *Cohort adjustment* include year of compulsory school graduation (11 levels) and year of observation (16 levels) as fixed-effects. *Demographic adjustment* include cohort covariates, sex, and immigrant status. *Mediation* covariates varied by outcome as follows: Income included days unemployed, presence of study benefits and disability pension respectively. Unemployment included disability pension and presence of study benefits. Disability pension included an indicator of being at least 19 years of age in 2003 or later due to an eligibility requirement implemented this year. *Comorbidity* includes indicators for a lifetime diagnosis of developmental disorder or intellectual disability. *Lifetime EA* includes indicators of lifetime secondary and tertiary educational attainment. | | | | | | | | | | |
