# Supplementary material for: Attention-deficit/hyperactivity disorder and occupational outcomes: The role of educational attainment, comorbid developmental disorders, and intellectual disability
Source: PLoS One. 2021 Mar 17;16(3):e0247724. doi: 10.1371/journal.pone.0247724 (PMC7968636; doi:10.1371/journal.pone.0247724)
Supplement: S1 Table — (DOCX) [file pone.0247724.s004.docx]

S1 Table. Analyses of nominal income

As the main analyses were made on the logarithmically transformed income variable where negative observations were excluded, analyses of the untransformed variable are included here for reference.

| **Associations between ADHD and nominal income, and the influence of intellectual disability/developmental disorders and lifetime educational attainment** | | | | |
| --- | --- | --- | --- | --- |
|  | **Cohort** | **Demographic and mediation** | **Demographic, mediation and comorbid ID/DD** | **Demographic, mediation and lifetime EA** |
| Income difference (euro) | -2,058  (-2,108, -2,007) | -1,640  (-1,689, -1,589) | -1,408  (-1,459, -1,357) | -1,234  (-1,284, -1,185) |
| See the methods section and the notes of Table 2 in the manuscript for details on the model. | | | | |
