# Supplementary material for: Attention-deficit/hyperactivity disorder and occupational outcomes: The role of educational attainment, comorbid developmental disorders, and intellectual disability
Source: PLoS One. 2021 Mar 17;16(3):e0247724. doi: 10.1371/journal.pone.0247724 (PMC7968636; doi:10.1371/journal.pone.0247724)
Supplement: S2 Table — (DOCX) [file pone.0247724.s005.docx]

S2 Table. Association between ADHD and graduation year from secondary/tertiary educations

|  | **Year of graduation** | |  |
| --- | --- | --- | --- |
| **Education** | **ADHD** | **Controls** | **Difference** |
| Secondary | 4.23 (4.21, 4.24) | 3.51 (3.50, 3.52) | 0.72 (0.70, 0.73) |
|  | Individuals with a lifetime tertiary education | | |
| Secondary^a^ | 3.60 (3.58, 3.63) | 3.23 (3.23, 3.24) | 0.37 (0.34, 0.40) |
| Terttiary | 9.28 (9.19, 9.36) | 8.34 (8.32, 8.36) | 0.93 (0.85, 1.02) |
| ^a^: Graduation from a secondary education among those with a tertiary education.  Associations have been estimated using linear regression and are adjusted for year of graduation from compulsory school (11 levels). Year of graduation is counted from compulsory school graduation as in the main analyses (0-15 years). | | | |
