# Supplementary material for: Attention-deficit/hyperactivity disorder and occupational outcomes: The role of educational attainment, comorbid developmental disorders, and intellectual disability
Source: PLoS One. 2021 Mar 17;16(3):e0247724. doi: 10.1371/journal.pone.0247724 (PMC7968636; doi:10.1371/journal.pone.0247724)
Supplement: S3 Table — (DOCX) [file pone.0247724.s006.docx]

S3 Table. Influence of educational attainment on occupational trajectories in ADHD

This table shows the results from the models using EA as a time-varying exposure, both crude and adjusted associations. In the accompanying manuscript, these models were used to produce Figure 1 and 2. In the table, “Level” represents difference between a period before and after an educational attainment, and “Trend” a yearly time trend for an educational attainment. For example, compared to the general population attaining a secondary education, individuals with ADHD on average had a 4 percent lower income (income ratio=0.96 [0.95, 0.98] in the period after the educational completion relative to the period before. During this period, the income trend for individuals with ADHD was not distinguishable (income ratio=1.00) from the general population (income ratio=1.00). Note that the association denoted “Level” for individuals with ADHD and compulsory EA is a main effect of (lifetime) ADHD. No compulsory level effect exists for controls since this is subsumed in the population trend in occupational outcomes.

| **Influence of EA on occupational trajectories in ADHD** | | | | | | |
| --- | --- | --- | --- | --- | --- | --- |
|  | **ADHD** | | | **Population** | | |
|  | **Educational attainment** | | | **Educational attainment** | | |
|  | **Compulsory** | **Secondary** | **Tertiary** | **Compulsory** | **Secondary** | **Tertiary** |
|  | **Income ratio** | | | | | |
|  | **Crude** | | | | | |
| **Level** | 0.93  (0.92, 0.94) | 0.94  (0.92, 0.96) | 1.31  (1.19, 1.45) | REF | 1.44  (1.44, 1.45) | 0.71  (0.71, 0.72) |
| **Trend** | 0.99  (0.99, 0.99) | 1.00  (0.99, 1.00) | 0.98  (0.97, 0.99) | REF | 0.98  (0.98, 0.98) | 1.03  (1.03, 1.03) |
|  | **Adjusted** | | | | | |
| **Level** | REF | 0.96  (0.95, 0.98) | 1.15  (1.07, 1.23) | REF | 1.28  (1.27, 1.28) | 0.80  (0.79, 0.80) |
| **Trend** | 0.99  (0.98, 0.99) | 1.00  (1.00, 1.01) | 0.98  (0.98, 0.99) | REF | 0.99  (0.99, 0.99) | 1.03  (1.03, 1.03) |
|  | **Unemployment days** | | | | | |
|  | **Crude** | | | | | |
| **Level** | 3.64  (3.32, 3.96) | 11.25  (10.03, 12.46) | -15.25  (-20.91, -9.59) | REF | -1.96  (-2.29, -1.62) | -13.83  (-14.13, -13.54) |
| **Trend** | 0.24  (0.10, 0.38) | -0.26  (-0.49, -0.04) | 1.06  (0.44, 1.68) | REF | -1.56  (-1.62, -1.49) | 0.58  (0.55, 0.62) |
|  | **Adjusted** | | | | | |
| **Level** | REF | 17.46  (16.20, 18.73) | -12.58  (-18.46, -6.69) | REF | 10.67  (10.34, 11.00) | -3.60  (-3.91, -3.28) |
| **Trend** | 1.14  (1.00, 1.28) | -1.20  (-1.42, -0.97) | 1.24  (0.59, 1.88) | REF | -1.99  (-2.05, -1.93) | 0.49  (0.45, 0.53) |
|  | **Disability pension, Odds-ratio; % risk-difference** | | | | | |
|  | **Crude** | | | | | |
| **Level** | 1.00  (1.00, 1.00) | 1.01  (1.00, 1.02) | 0.98  (0.96, 1.01) | REF | 0.98  (0.97, 0.98) | 0.99  (0.99, 0.99) |
| **Trend** | 1.03  (1.02, 1.03) | 0.99  (0.99, 0.99) | 0.99  (0.99, 0.99) | REF | 1.00  (1.00, 1.00) | 1.00  (1.00, 1.00) |
|  | **Adjusted** | | | | | |
| **Level** | REF | 2.21; 0.19  (1.69, 2.89) | 1.13; 0.03  (0.18, 6.97) | REF | 0.15; -0.43  (0.13, 0.17) | 0.05; -0.62  (0.02, 0.08) |
| **Trend** | 1.24; 0.05  (1.19, 1.29) | 0.88; -0.03  (0.84, 0.92) | 1.00; -0.00  (0.85, 1.18) | REF | 1.09; 0.02  (1.07, 1.12) | 1.06; 0.01  (1.00, 1.13) |
| **REF:** Reference. As (virtually) all individuals graduate compulsory school at the start of follow-up, the only diﬀerence estimated here is the yearly trend speciﬁc to ADHD. **Level:** The associated diﬀerence between the period after an educational completion compared to the period before (i.e., 1 in the years after an EA, 0 before), by the presence of ADHD (e.g., 1 when ADHD and the year is after secondary EA). **Trend:** Secondary and Tertiary columns denote the interactions between a yearly trend (0-15), ADHD and each educational attainment respectively (i.e., 1 the years after an EA, 0 before).  **Model speciﬁcation:** All models included year of observation as a population ﬁxed-eﬀect (16 levels). The crude models included indicators for year of compulsory school graduation (11 levels), and an indicator for a lifetime diagnosis of ADHD (1 when true, 0 otherwise). The adjusted models included individual ﬁxed-eﬀects (within individual model). Covariates in the adjusted models varied by outcome follows. Income: Indicators of study benefits and disability pension, days unemployed, and a one year lag of log(income) (i.e., income at t regressed on income at t−1). Unemployment: Indicators of study income and disability pension. Disability pension: Unemployment, study beneﬁts, and an indicator of being 19 years of age or older in 2003 as an eligibility requirement was implemented in this year | | | | | | |
