# Supplementary material for: Attention-deficit/hyperactivity disorder and occupational outcomes: The role of educational attainment, comorbid developmental disorders, and intellectual disability
Source: PLoS One. 2021 Mar 17;16(3):e0247724. doi: 10.1371/journal.pone.0247724 (PMC7968636; doi:10.1371/journal.pone.0247724)
Supplement: S1 Fig — (DOCX) [file pone.0247724.s007.docx]

S1 Fig. Sensitivity analyses of income trajectories

Figures S1A and S1B reproduces Figure 1 and 2 in the manuscript, but using estimates from a model with individual fixed effects (within individual analysis) and no lagged value of income, and one model without individual fixed effect and a lagged value of income.

| **Figure S1A. Influence of ADHD on occupational trajectories by observed lifetime educational attainment** | | |
| --- | --- | --- |
| **Observed lifetime educational attainment** | | |
| **Compulsory** | **Secondary** | **Tertiary** |
| 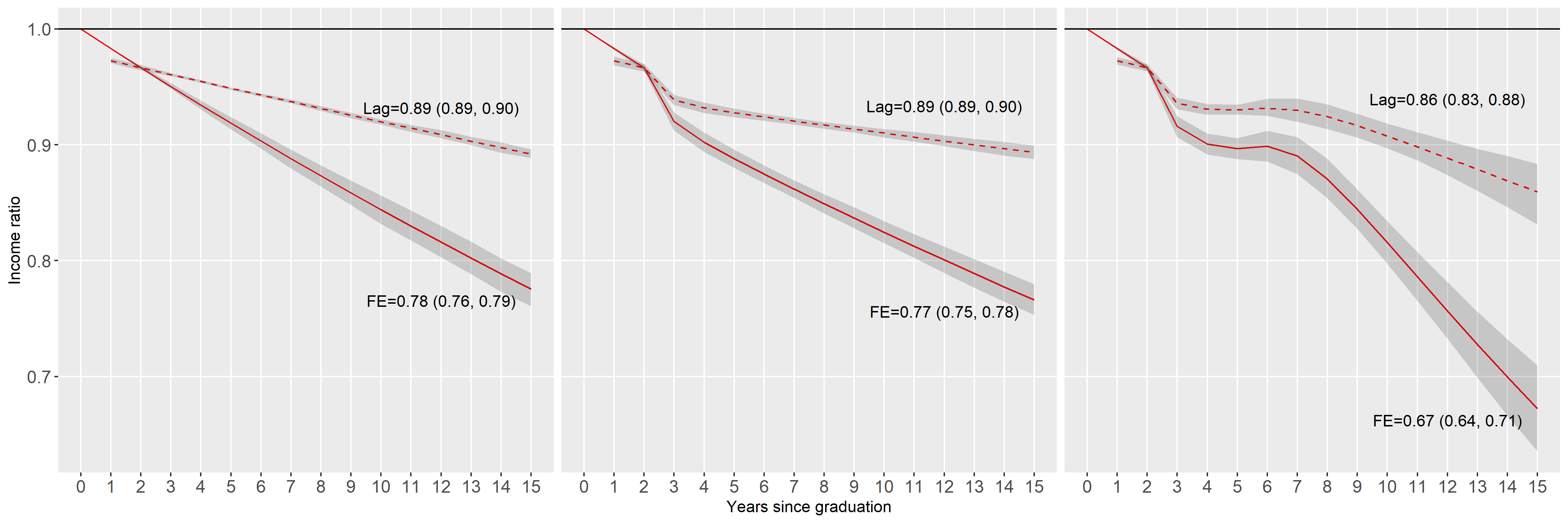 | | |
| **Red dashed line (Lag)**: Model including a lagged value of income, but no individual fixed effects. **Red solid line (FE):** Model including an individual fixed-effects, but no lagged value of income. Estimates in plot areas represent the estimated associations and 95% confidence intervals at the final point of observation (i.e., year 15). Shaded areas indicate 95 percent, quantile based, bootstrapped confidence intervals. For details on the model, see manuscript. | | |

| **Figure S1B. Influence of educational attainment on occupational trajectories in ADHD** | |
| --- | --- |
| **Secondary relative to compulsory** | **Tertiary relative to secondary** |
| **Individual fixed-effects** | |
| 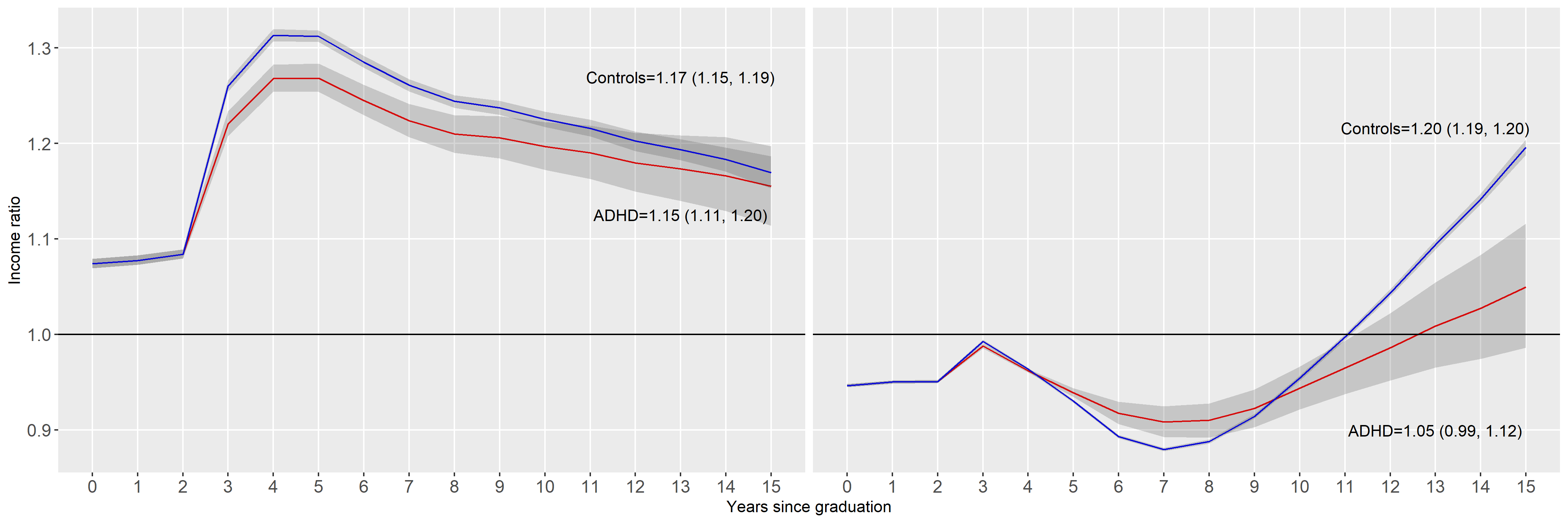 | |
| **Lagged income** | |
| 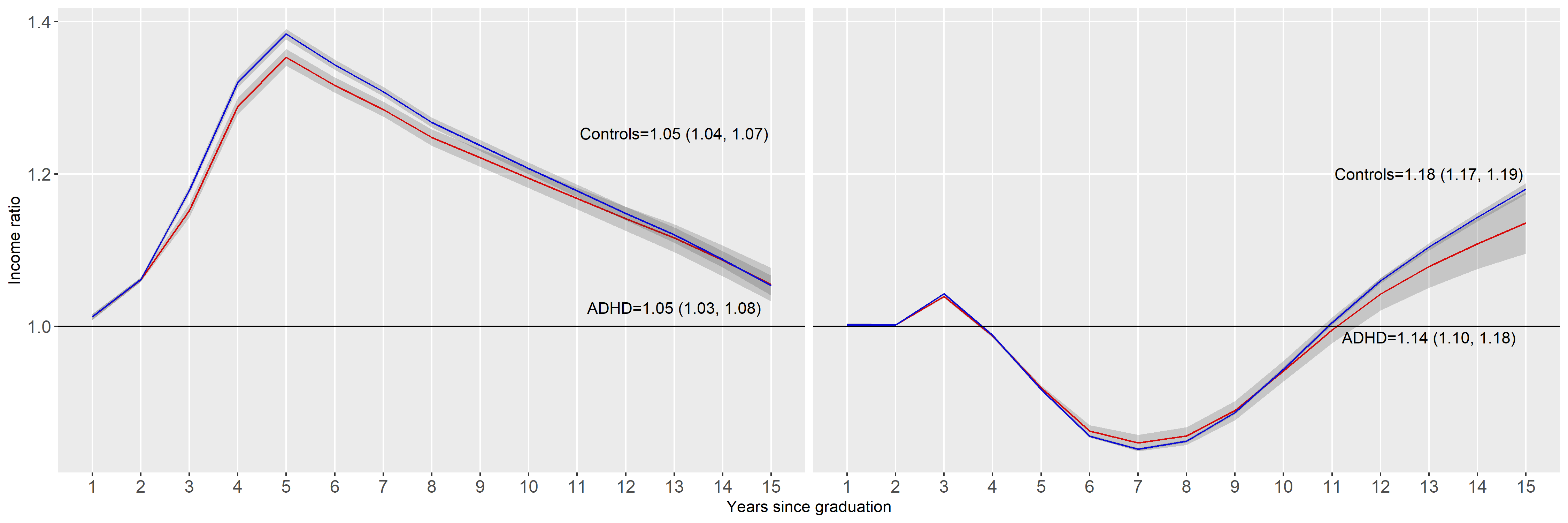 | |
| **Red line:** Individuals with a lifetime diagnosis of ADHD. **Blue line**: Controls. **Individual fixed-effects:** Model including an individual fixed-effects, but no lagged value of income. **Lagged income:** Model including a lagged value of income, but no individual fixed effects.  Estimates in plot areas represent the estimated associations and 95% confidence intervals at the final point of observation (i.e., year 15). Shaded areas indicate 95 percent, quantile based, bootstrapped confidence intervals. For more details on the model, see manuscript. | |
